# Supplementary material for: Trajectory of weight regain after cessation of GLP-1 receptor agonists: a systematic review and nonlinear meta-regression
Source: eClinicalMedicine. 2026 Mar 4;93:103796. doi: 10.1016/j.eclinm.2026.103796 (PMC13043475; doi:10.1016/j.eclinm.2026.103796)
Supplement: Supplementary Tables and Figures [file mmc1.docx]

**Supplementary material**

Trajectory of weight regain after cessation of GLP-1 receptor agonists: a systematic review and nonlinear meta-regression

**Table of contents**

[1. Search terms 2](#_5m19f0vibdh7)

[2. Summary of included studies 3](#_na4vw3q0z4ub)

[3. Risk of bias assessments 7](#_7m7bsxol6qlh)

[4. Sensitivity analyses 11](#_5txekza698e3)

[5. GRADE summary of findings 20](#_unex3tpopv3q)

[References 21](#_m4rzsqmtjjj1)

# 1. Search terms

#

Ovid MEDLINE(R) and Epub Ahead of Print, In-Process,

In-Data-Review & Other Non-Indexed Citations, Daily and Versions

<1946 to August 28, 2025>

1 exp Glucagon-Like Peptide 1/ or (glucagon-like peptide 1 or GLP-1 or

GLP1).ti,ab,kw,kf.

2 (agonist* or analog*).ti,ab,kw,kf.

3 (GLP-1RA* or GLP1RA*).ti,ab,kw,kf.

4 (liraglutide or semaglutide or tirzepatide or exenatide or

lixisenatide or albiglutide or dulaglutide or efpeglenatide or beinaglutide

or taspoglutide or pegapamodutide or mazdutide or retatrutide or

danuglipron or orforglipron).ti,ab,kw,kf.

5 (1 and 2) or 3 or 4

6 (weight adj3 (gain* or regain* or los* or maint* or chang* or

difference or increas* or decreas* or reduc* or declin* or trajectory or

control* or normali* or fluctuat* or r?se* or rebound* or recover* or

restor* or relaps*)).ti,ab,kw,kf.

7 randomized controlled trial.pt. or (random* and

placebo).ti,ab,kw,kf.

8 week*.ti,ab,kw,kf.

9 7 and 8

10 (cohort or observational or retrospective or

prospective).ti,ab,kw,kf.

11 (withdraw* or discontin* or ceas* or cessation or deprescri* or

stop* or termin* or finish* or paus* or "not continu*" or suspend* or

conclud*).ti,ab,kw,kf.

12 10 and 11

13 5 and 6 and (9 or 12)

# 2. Summary of included studies

**Table S1. Included studies**

| **Study** | **Design** | **Comorbidities (except obesity)** | **Total participants (all arms)** | **GLP-1RA** | **Cotreatments** | **Treatment length (weeks)** | **Post-treatment follow-up length (weeks)** |
| --- | --- | --- | --- | --- | --- | --- | --- |
| Altintas Dogan et al. (2022)[^1^](https://www.zotero.org/google-docs/?WjfLMq) | RCT | COPD | 40 | liraglutide | - | 40 | 4 |
| Apperloo et al. (2025)[^2^](https://www.zotero.org/google-docs/?DbeC3N) | RCT | CKD | 101 | semaglutide | ACE inhibitors, SGLT2 inhibitors | 24 | 4 |
| Armstrong et al. (2016)[^3^](https://www.zotero.org/google-docs/?EQWzZ1) | RCT | MASH | 52 | liraglutide | lifestyle advice | 48 | 12 |
| Aronne et al. (2024)[^4^](https://www.zotero.org/google-docs/?Xy4kIH) | RCT | - | 670 | tirzepatide | lifestyle advice | 36 | 52 |
| Asano et al. (2023)[^5^](https://www.zotero.org/google-docs/?WcVIUk) | RCT | T2DM | 16 | cotadutide | - | 10 | 4 |
| Barnett et al. (2007)[^6^](https://www.zotero.org/google-docs/?ErQrOU) | RCT  (crossover) | T2DM | 138 | exenatide | metformin/sulfonylurea, insulin glargine after stopping exenatide | 16 | 16 |
| Bartelt et al. (2024)[^7^](https://www.zotero.org/google-docs/?TVpjEA) | retrospective cohort | - | 38,007 | semaglutide, liraglutide | - | variable | 52 |
| Bunck et al. (2011)[^8^](https://www.zotero.org/google-docs/?nw3pNp) | RCT | T2DM | 69 | exenatide | metformin | 52, 104 | 12, 12 |
| Chen et al. (2024)[^9^](https://www.zotero.org/google-docs/?zcs2IO) | RCT | - | 427 | beinaglutide | lifestyle advice | 16 | 12 |
| D'Alessio et al. (2014)[^10^](https://www.zotero.org/google-docs/?eS78I7) | RCT  (crossover) | T2DM | 978 | liraglutide | metformin ± sulfonylureas, insulin glargine after stopping liraglutide | 24 | 24 |
| Davies et al. (2015)[^11^](https://www.zotero.org/google-docs/?SBGKg5) | RCT | T2DM | 846 | liraglutide | lifestyle advice + metformin ± sulfonylureas, glitazone | 56 | 12 |
| Dusilová et al. (2024)[^12^](https://www.zotero.org/google-docs/?uPDmma) | RCT  (crossover) | MASLD | 16 | semaglutide | dietary intervention continued after stopping semaglutide | 16 | 16 |
| Enebo et al. (2021)[^13^](https://www.zotero.org/google-docs/?8lBbO9) | RCT | - | 96 | semaglutide | cagrilinitide or placebo | 20 | 5 |
| Ferjan et al. (2017)[^14^](https://www.zotero.org/google-docs/?Bwprdv) | RCT | PCOS | 24 | liraglutide | metformin or metformin + sitagliptin after stopping liraglutide | 12 | 12 |
| Ferrari et al. (2020)[^15^](https://www.zotero.org/google-docs/?1PdYvu) | retrospective cohort | - | 93 | liraglutide | lifestyle advice | variable | variable |
| Fineman et al. (2011)[^16^](https://www.zotero.org/google-docs/?Yp8CHY) | RCT | T2DM | 107 | exenatide | metformin + lifestyle advice | 15 | 12 |
| Frias et al. (2022)[^17^](https://www.zotero.org/google-docs/?DZbwvz) | RCT | T2DM | 406 | efpeglenatide | lifestyle advice | 56 | 6 |
| Garcia de Lucas and Olalla Sierra (2017)[^18^](https://www.zotero.org/google-docs/?JjFtNe) | prospective cohort | T2DM | 13 | liraglutide, lixisenatide, exenatide | canagliflozin | 36 | 26 |
| Gibbons et al. (2021)[^19^](https://www.zotero.org/google-docs/?rNyI2r) | RCT  (crossover) | T2DM | 15 | semaglutide | metformin + lifestyle advice | 12, 12 | 8, 8 |
| Jastreboff et al. (2025)[^20^](https://www.zotero.org/google-docs/?tjQZt1) | RCT | - | 1,032 | tirzepatide | lifestyle advice | 176 | 17 |
| Jensen et al. (2024)[^21^](https://www.zotero.org/google-docs/?W1UKRc) | RCT | - | 195 | liraglutide | low-calorie diet prior to starting liraglutide + lifestyle advice during treatment | 52 | 52 |
| Jensterle et al. (2024)[^22^](https://www.zotero.org/google-docs/?My7Uzu) | prospective cohort | PCOS | 25 | semaglutide | metformin + lifestyle advice | 16 | 104 |
| Ji et al. (2023)[^23^](https://www.zotero.org/google-docs/?klkxn4) | RCT | - | 248 | mazdutide | - | 24 | 12 |
| Ji et al. (2025)[^24^](https://www.zotero.org/google-docs/?RVuH9Q) | RCT | various weight-related coexisting conditions | 664 | ecnoglutide | lifestyle advice | 48 | 7 |
| Ji et al. (2025)[^25^](https://www.zotero.org/google-docs/?25DWLS) | RCT | various weight-related coexisting conditions | 610 | mazdutide | lifestyle advice | 48 | 12 |
| Khoo et al. (2019)[^26^](https://www.zotero.org/google-docs/?Ibw6Ka) | RCT | MASLD/MASH | 30 | liraglutide | lifestyle advice | 26 | 26 |
| Kubota et al. (2023)[^27^](https://www.zotero.org/google-docs/?PUuPhK) | prospective cohort | T2DM | 9 | tirzepatide | - | 52 | 104 |
| Lau et al. (2021)[^28^](https://www.zotero.org/google-docs/?F1y7Pz) | RCT | - | 706 | liraglutide | lifestyle advice | 26 | 6 |
| le Roux et al. (2017)[^29^](https://www.zotero.org/google-docs/?RnOvkJ) | RCT | prediabetes | 2,254 | liraglutide | lifestyle advice | 160 | 12 |
| McGowan et al. (2024)[^30^](https://www.zotero.org/google-docs/?kHmi9h) | RCT | prediabetes | 207 | semaglutide | lifestyle advice | 52 | 28 |
| McInnes et al. (2023)[^31^](https://www.zotero.org/google-docs/?zO73Fh) | RCT | T2DM | 160 | lixisenatide | insulin glargine + metformin + lifestyle advice | 12 | 52 |
| McKenzie and Athinarayanan (2024)[^32^](https://www.zotero.org/google-docs/?XoXqq7) | retrospective cohort | T2DM | 308 | unspecified | carbohydrate restricted nutrition therapy continued after stopping GLP-1RA | variable | 52 |
| Montvida et al. (2017)[^33^](https://www.zotero.org/google-docs/?wo9Pyt) | retrospective cohort | T2DM | 66,583 | unspecified | - | variable | variable |
| Moolla et al. (2025)[^34^](https://www.zotero.org/google-docs/?WfKOCM) | RCT | MASLD | 30 | liraglutide | - | 12 | 12 |
| O'Neil et al. (2018)[^35^](https://www.zotero.org/google-docs/?Cfw8MS) | RCT | - | 957 | semaglutide, liraglutide | lifestyle advice | 52 | 7 |
| Punthakee et al. (2024)[^36^](https://www.zotero.org/google-docs/?HTCLhQ) | RCT | T2DM | 159 | liraglutide | insulin degludec + metformin + lifestyle advice | 16 | 52 |
| Rosenstock et al. (2010)[^37^](https://www.zotero.org/google-docs/?taZGa7) | RCT | - | 152 | exenatide | lifestyle advice | 24 | 4 |
| Rubino et al. (2021)[^38^](https://www.zotero.org/google-docs/?dT9f2u) | RCT | - | 803 | semaglutide | lifestyle advice | 20 | 48 |
| Sanyal et al. (2024)[^39^](https://www.zotero.org/google-docs/?esq84O) | RCT | MASLD | 98 | retatrutide | lifestyle advice | 48 | 4 |
| Seier et al. (2025)[^40^](https://www.zotero.org/google-docs/?iq5vzR) | prospective cohort | - | 2,694 | semaglutide | weight-management programme | variable | 26 |
| Siskind et al. (2020)[^41^](https://www.zotero.org/google-docs/?Mg9Jmd) | RCT | clozapine-associated obesity | 28 | exenatide | clozapine | 24 | 52 |
| Svensson et al. (2019)[^42^](https://www.zotero.org/google-docs/?zR38yW) | RCT | clozapine-associated obesity | 103 | liraglutide | clozapine/olanzapine | 16 | 52 |
| Touzot et al. (2025)[^43^](https://www.zotero.org/google-docs/?Jbr5AF) | prospective non-randomised | T2DM | 18 | liraglutide | - | 26 | 12 |
| Varanasi et al. (2011)[^44^](https://www.zotero.org/google-docs/?b25Hzr) | retrospective cohort | T2DM | 141 | exenatide | lifestyle advice | variable | 26 |
| Wadden et al. (2013)[^45^](https://www.zotero.org/google-docs/?sYIuLK) | RCT | - | 422 | liraglutide | low-calorie diet prior to starting liraglutide | 56 | 12 |
| Wilding et al. (2022)[^46^](https://www.zotero.org/google-docs/?OmYc2m) | RCT | - | 327 | semaglutide | lifestyle advice | 68 | 52 |
| Yu et al. (2022)[^47^](https://www.zotero.org/google-docs/?LYc0zE) | retrospective cohort | - | 157 | liraglutide | - | variable | variable |
| Zhou et al. (2023)[^48^](https://www.zotero.org/google-docs/?nFQDMr) | prospective cohort | T2DM | 98 | liraglutide | lifestyle advice | 12 | variable |

#

# 3. Risk of bias assessments

#
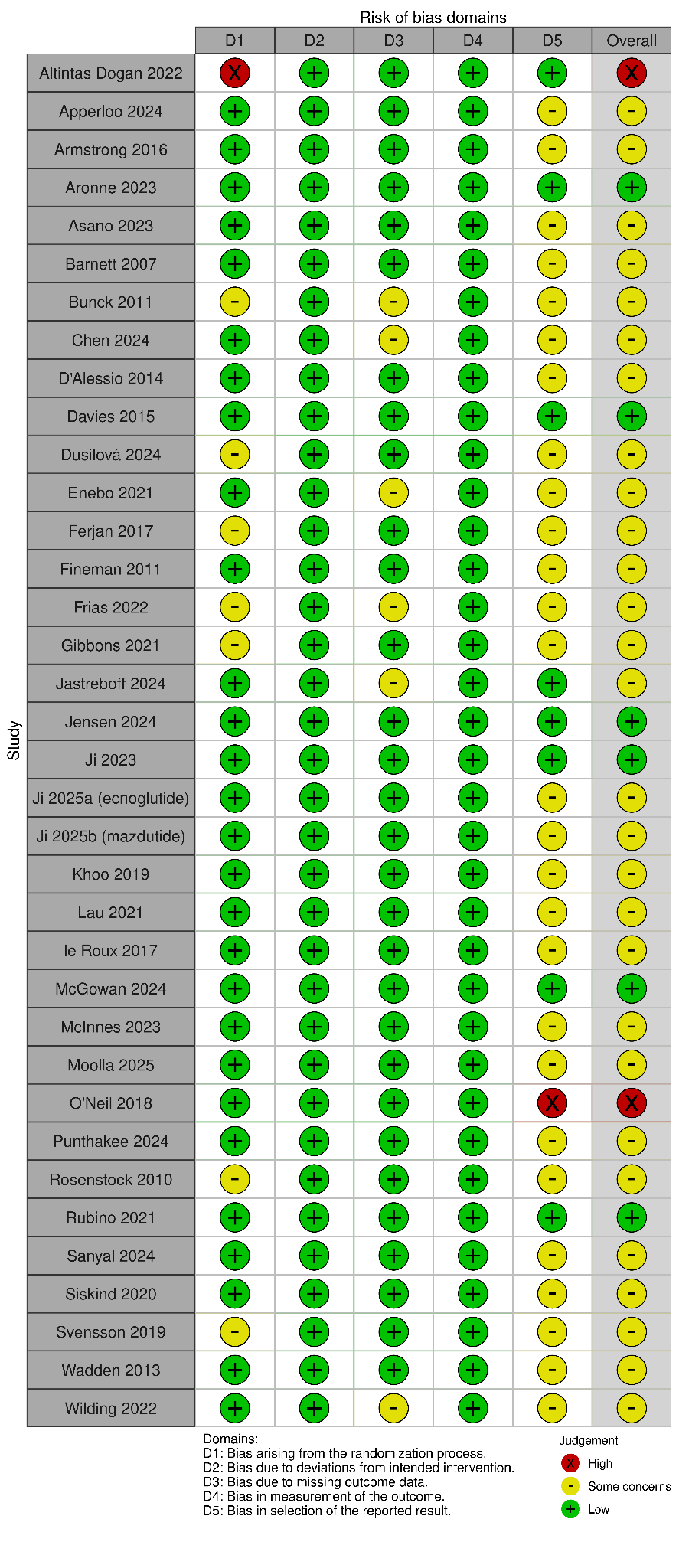


**Figure S1. RoB 2 traffic light plot of randomised controlled trials (weight).**

**
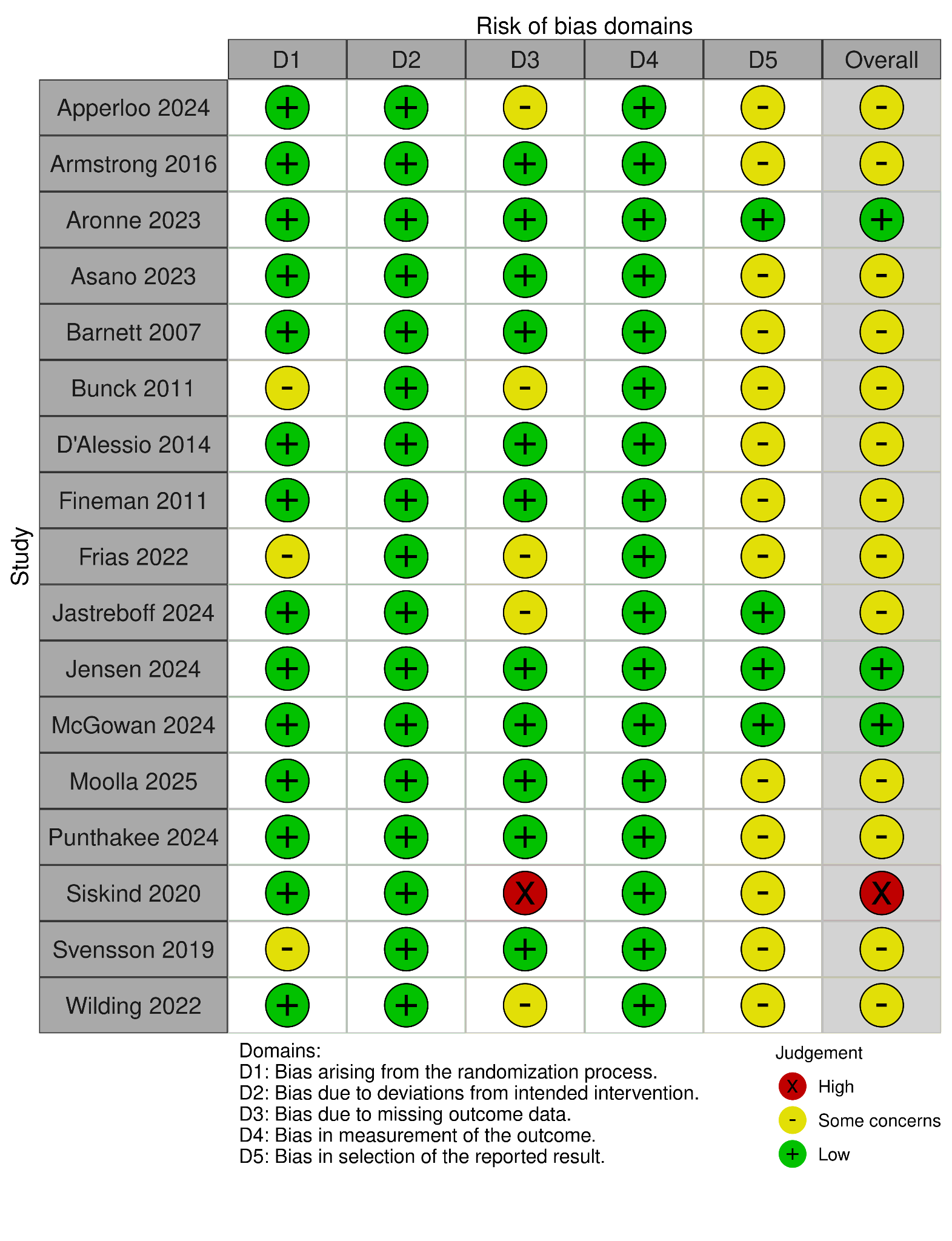
**

**Figure S2. RoB 2 traffic light plot of randomised controlled trials (HbA1c).**


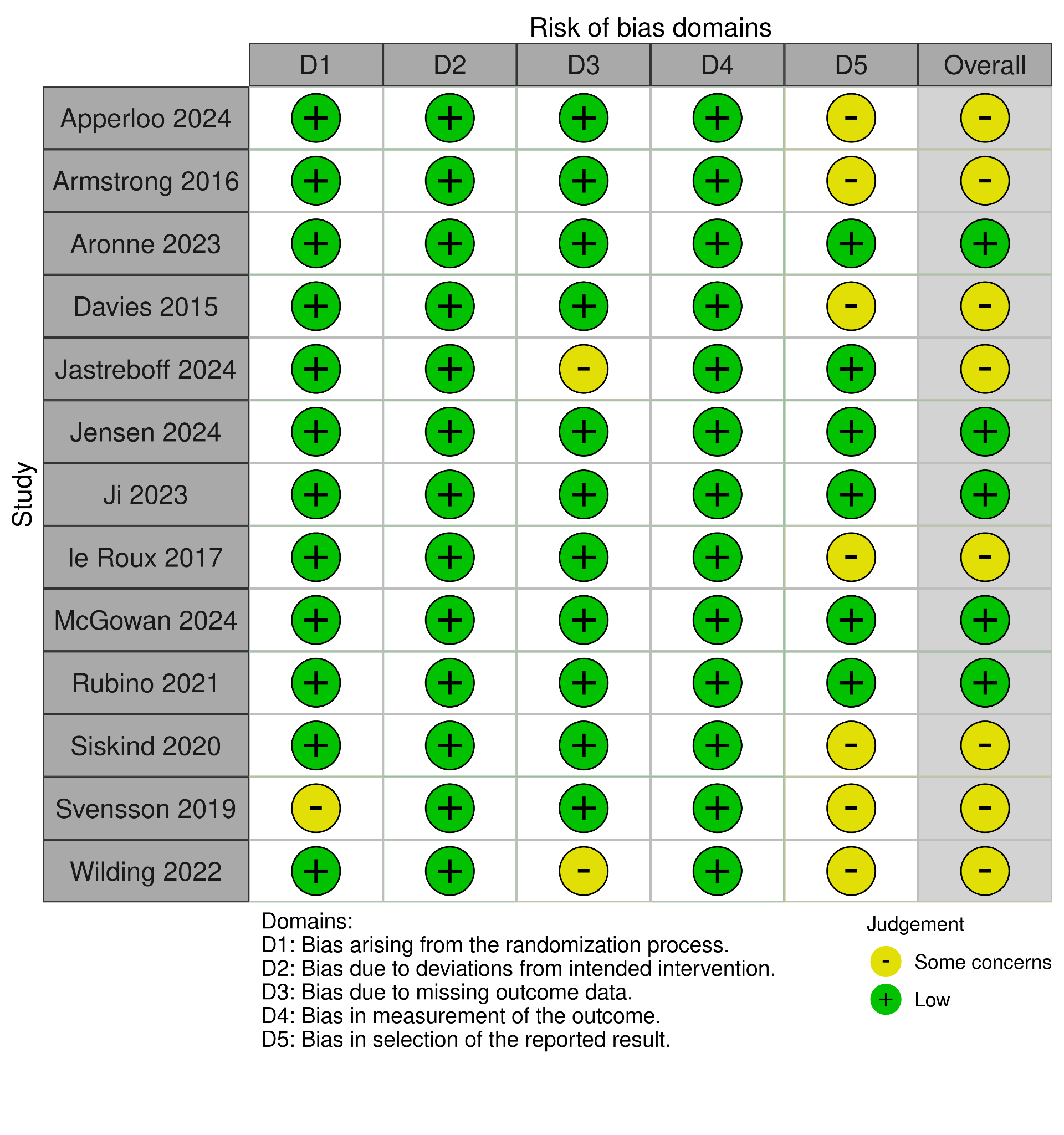


**Figure S3. RoB 2 traffic light plot of randomised controlled trials (systolic blood pressure).**

#


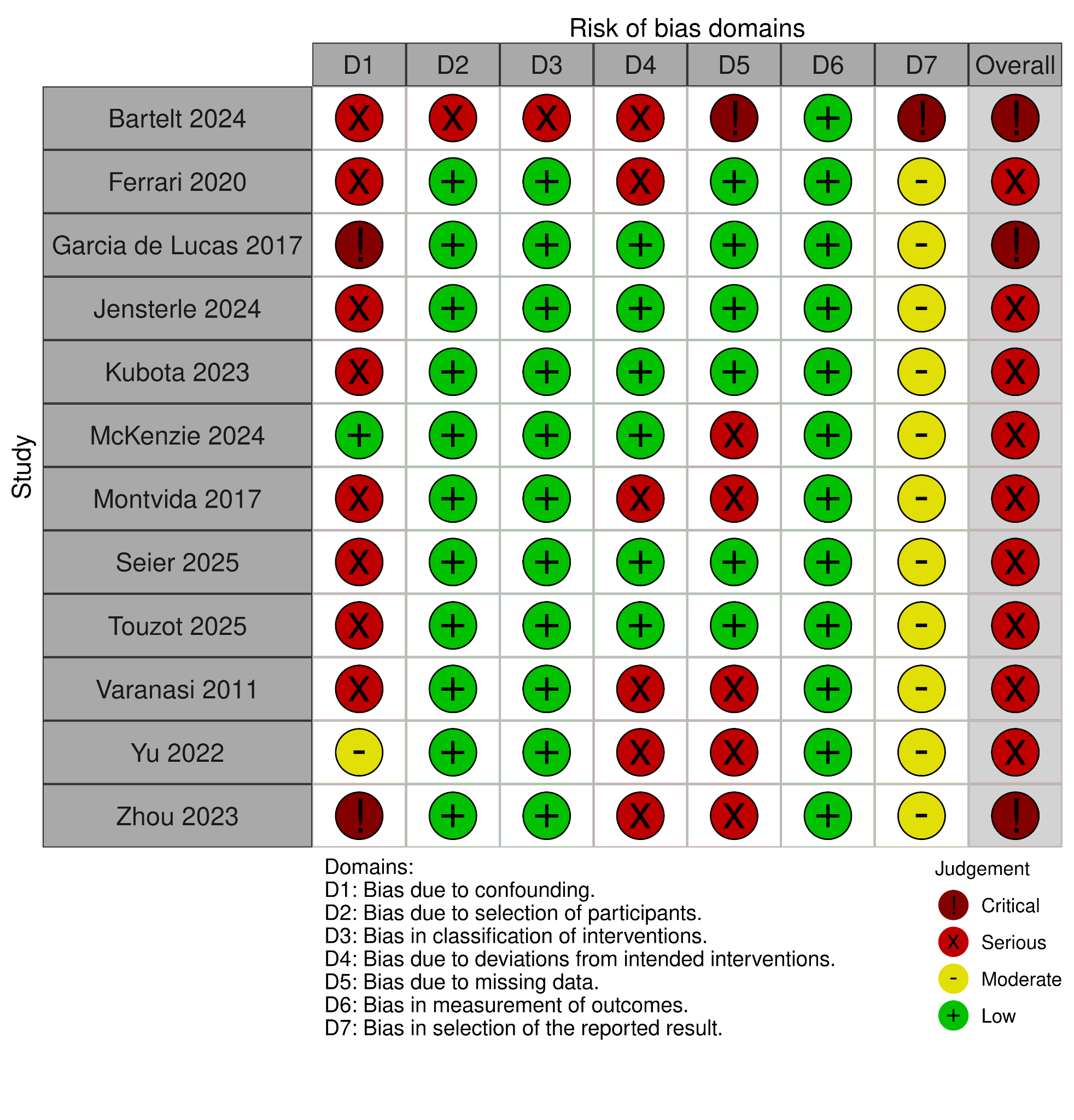


**Figure S4. ROBINS-I traffic light plot of non-randomised studies (weight).**

#


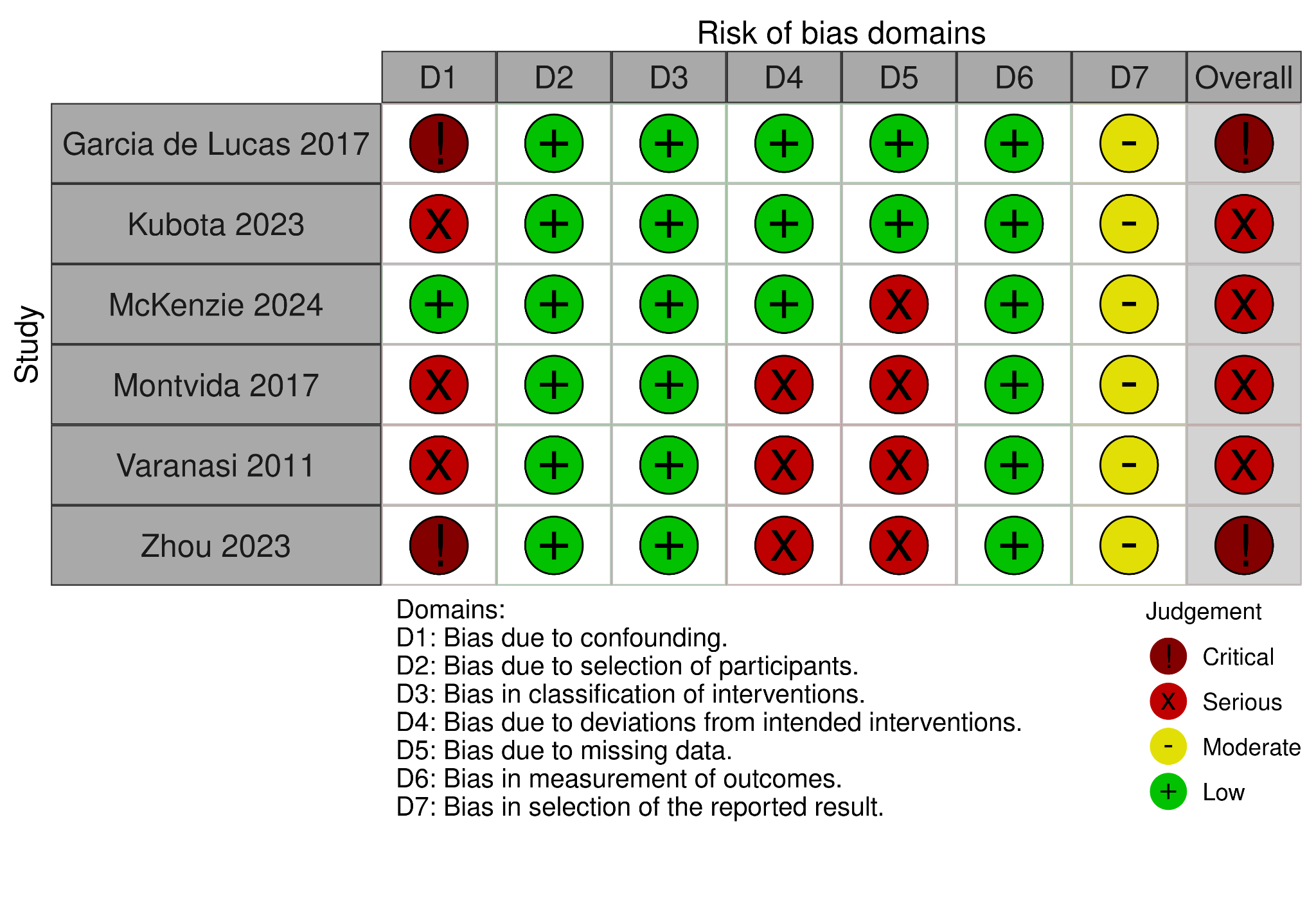


**Figure S5. ROBINS-I traffic light plot of non-randomised studies (HbA1c).**

#

#
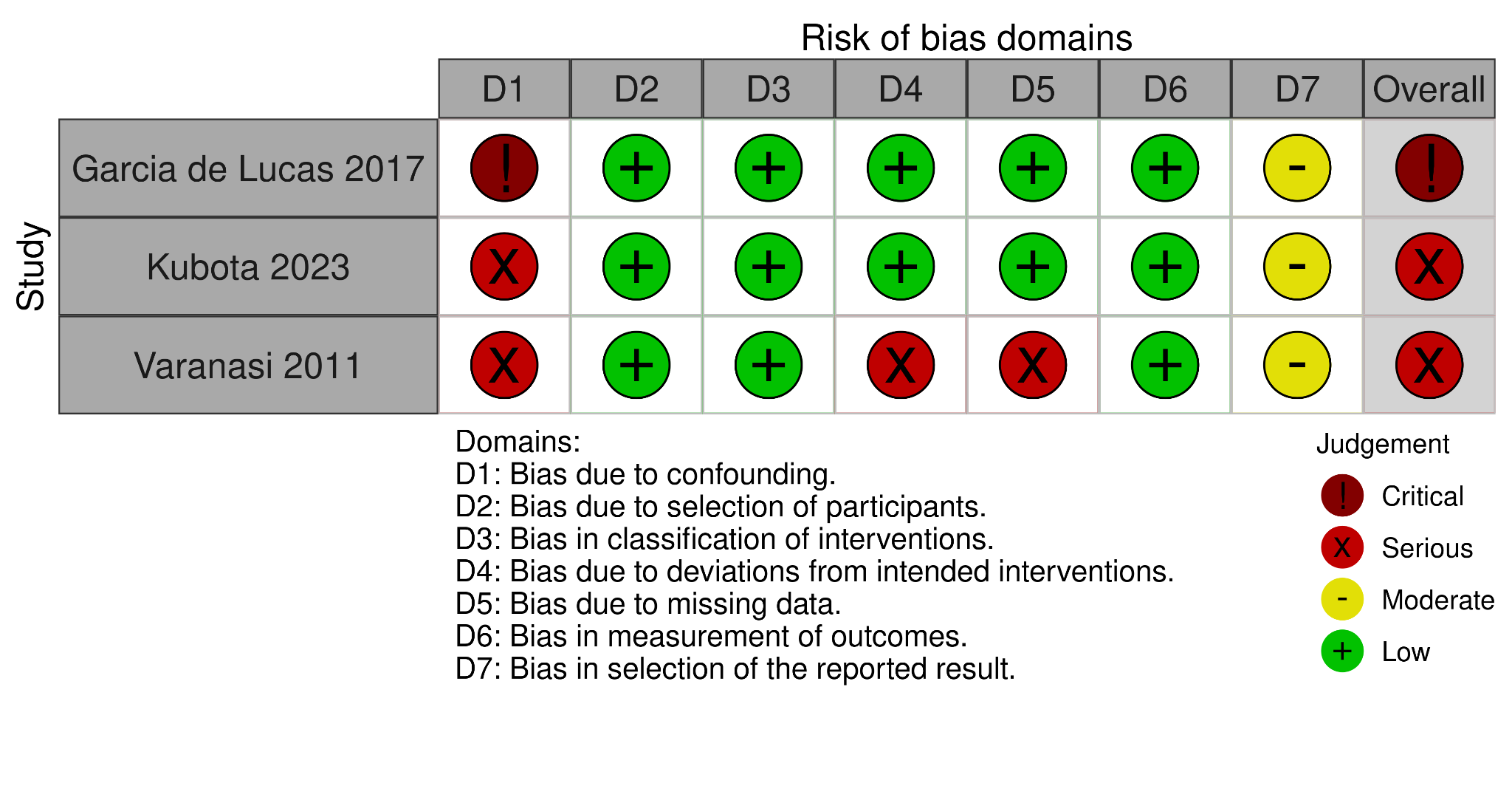


**Figure S6. ROBINS-I traffic light plot of non-randomised studies (systolic blood pressure).**

#

# 4. Sensitivity Analyses

#

**A. Bounded model with logit transform**

Biologically, percentage weight regain is expected to be bounded between 0-100%, however the exponential recovery model could theoretically produce predictions exceeding this (although this did not occur in our original model). In addition, residuals may not be normally distributed near bounds. To assess the impact of these concerns, we refitted the model using the logit-transformed proportion of weight gained, and back-transformed the resulting curves to the original percentage weight gain scale (figure S7). The logit transformation maps the proportion regained onto the real line, allowing the model to operate in an unbounded space while ensuring that, after back-transformation, all predicted values lie strictly within the 0-100% range. The spread of residuals with the back-transformed logit model showed no improvement over the original model (figure S8). In addition, estimated parameters (A = 0.803, k = 0.0281) and the back-transformed trajectory closely resembled the original model, supporting the robustness of our findings. Residuals vs fits plots are shown in figure S9.


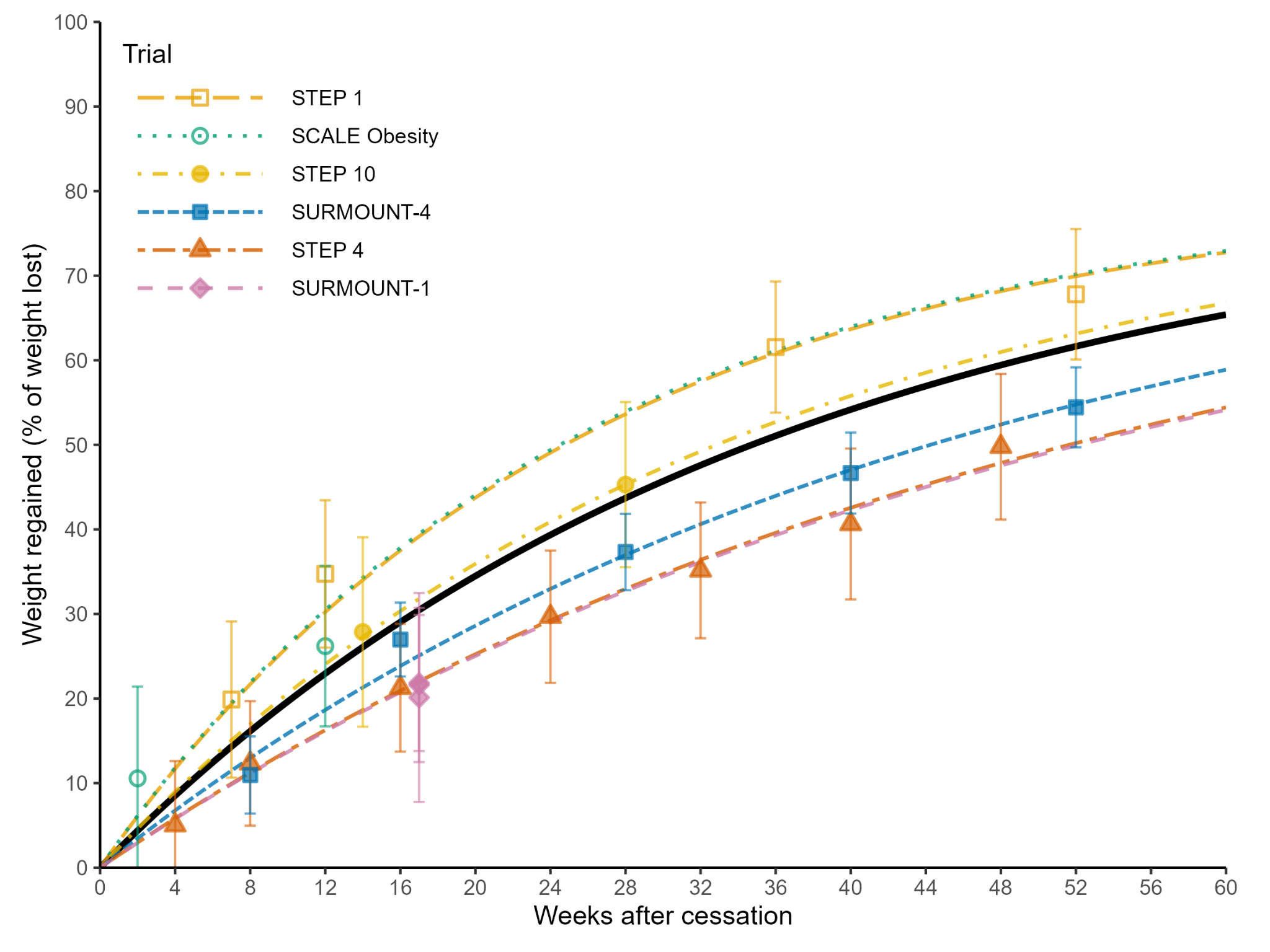


**Figure S7. Back-transformed predictions from the logit-transformed exponential recovery model.** Predictions were generated on the logit scale and converted back to percentage weight regained to ensure values were bounded within the 0-100% range.


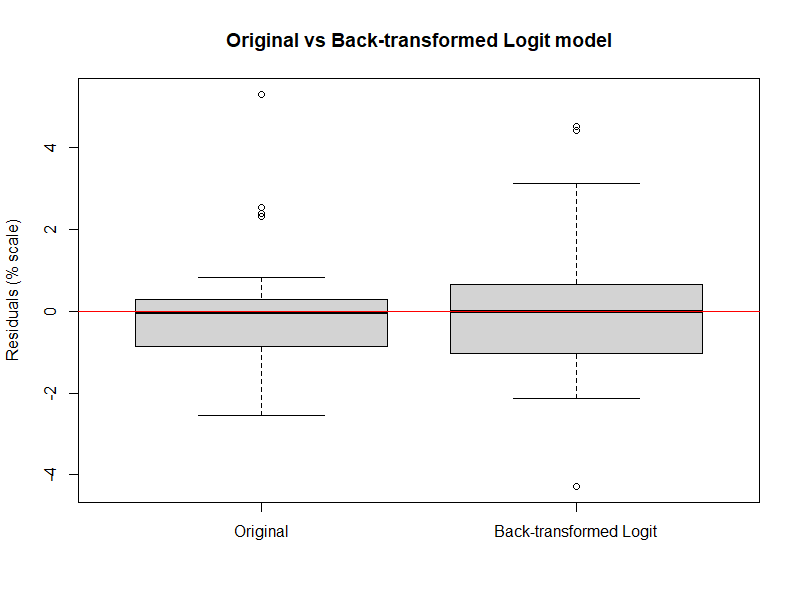


**Figure S8. Distribution of residuals on the percentage scale in the original and back-transformed logit models.**


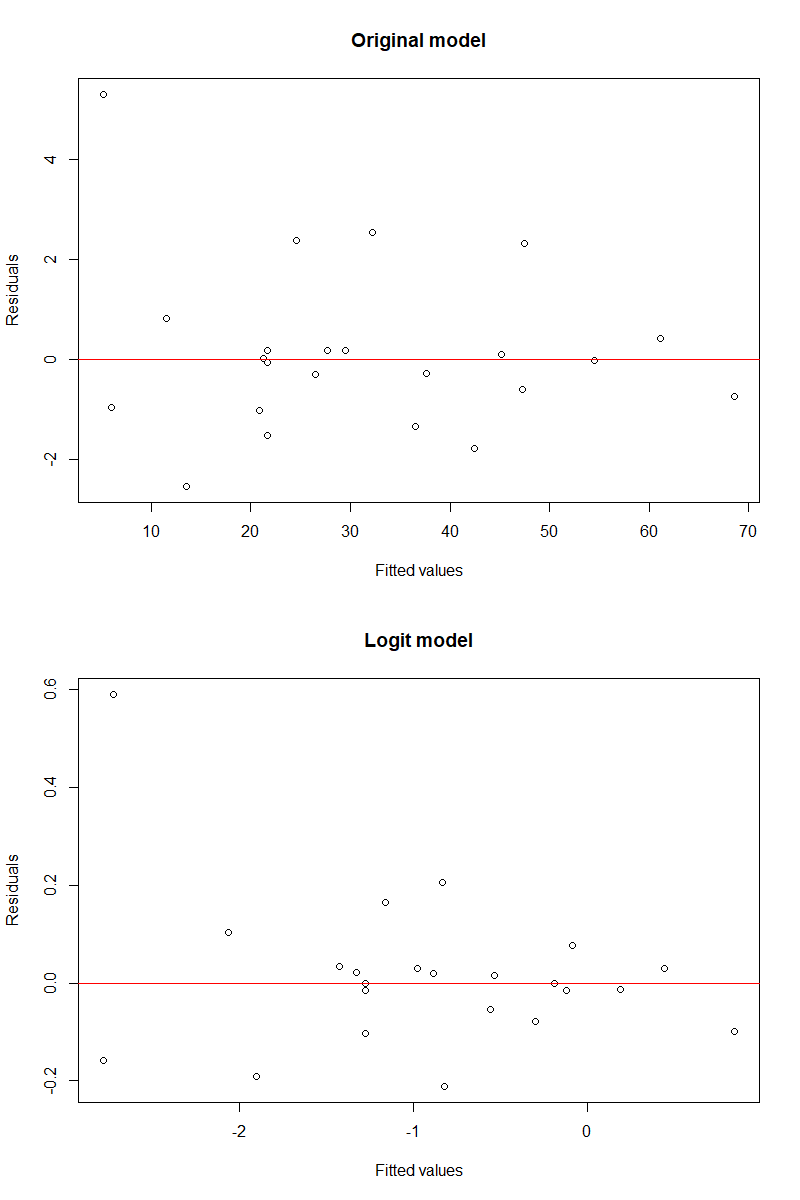


**Figure S9. Residuals vs fits plots for the original and logit-transformed models.**

**B. Alternative random effects**

In the original model, random effects were applied to *k* only. We tested two alternative model specifications: one model with random effects on *A* rather than *k*, and another model with random effects applied to both *A* and *k*. The model with random effects on *A* only showed a visually poorer fit to the data (figure S10) and a higher Akaike Information Criterion (AIC) and Bayesian Information Criterion (BIC) than the original model (table S2). The model with random effects on both A and k resulted in the optimiser producing boundary fits, where the random effect variances of one of the parameters consistently collapsed to near zero, resulting in a model that was effectively the same as one of the previous two models.


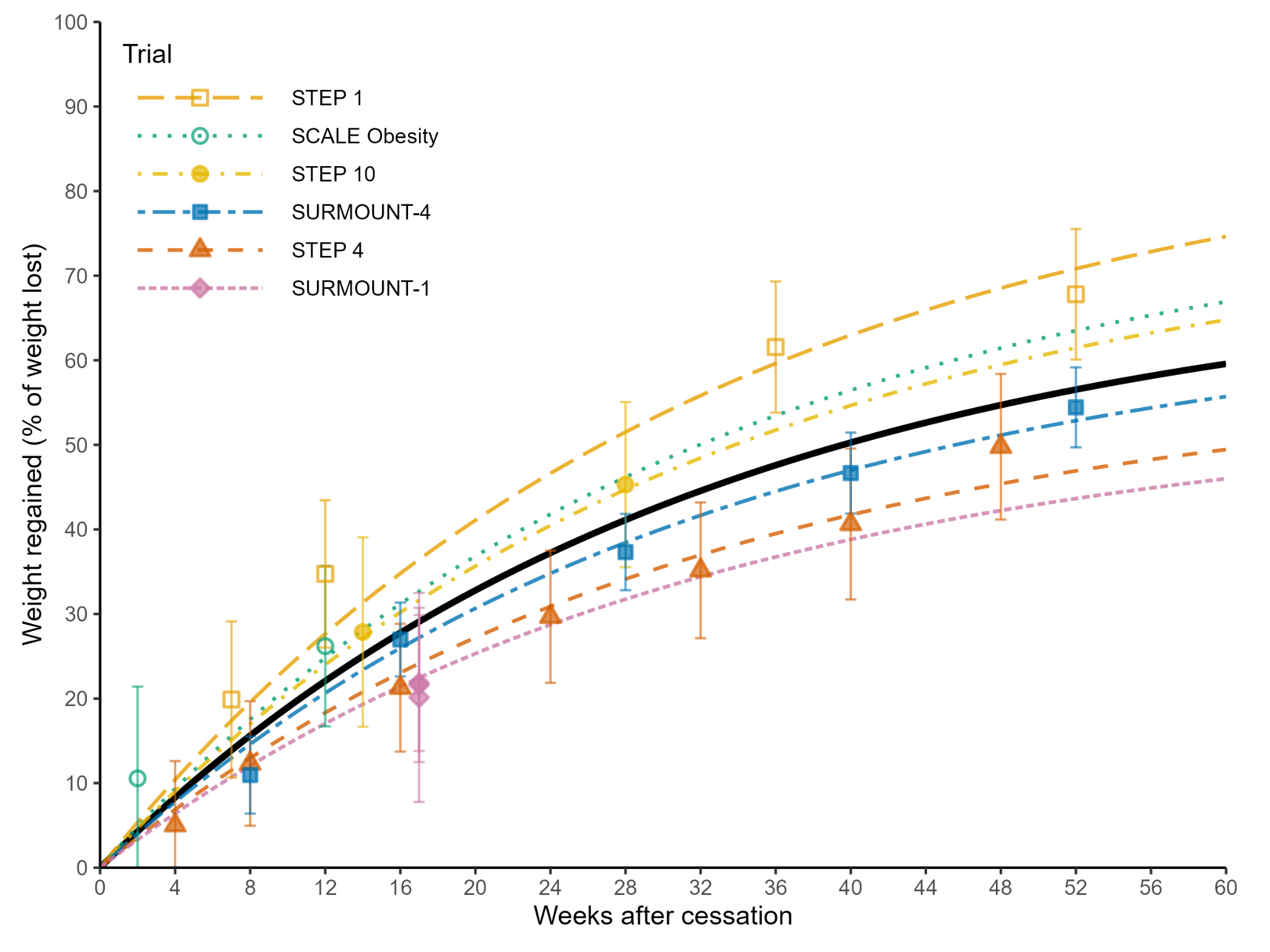


**Figure S10. Alternative model with random effects on *A* rather than *k*.**

| **Model** | **AIC** | **BIC** |
| --- | --- | --- |
| Random effects on *k* | 126.6535 | 131.1955 |
| Random effects on *A* | 138.7014 | 143.2434 |

**Table S2. AIC and BIC of alternative random-effect specifications.** The model with random effects on k showed a lower AIC and BIC, indicating it was a better fit.

**C. Time point correlation**

To account for potential within-arm correlation, we compared models assuming independent errors (iid), continuous-time AR(1) correlation (CAR(1)), and compound symmetry (CS) . Estimates of A and k were stable across structures. Under iid, A=76.2% (95% CI 69.3–83.1) and k=0.0270 per week (95% CI 0.0189–0.0351); under CAR(1), A=72.7% (95% CI 56.3–89.0) and k=0.0298 per week (95% CI 0.0178–0.0419); and under CS, A=74.0% (95% CI 63.4–84.6) and k=0.0276 per week (95% CI 0.0191–0.0362). Model fit was similar (AIC 132 with CAR(1) vs 141 with CS). For CAR(1), the estimated decay parameter was ϕ=0.98, corresponding to a correlation of 0.38 between residuals one week apart. Under CS, the common within-arm correlation was ρ=0.82.

SURMOUNT-1 included multiple doses at the same time point. For comparability, all sensitivity analyses (iid, CAR(1), CS) were fit with arm-level grouping (study × drug × dose) to ensure unique timepoints for correlation structures. This differs from the main iid model, which used study-level grouping.

**D. Monte Carlo simulation of digitisation error**

Data used in the model were extracted via digitisation in WebPlotDigitizer. We used Monte Carlo simulation to assess the effect of digitisation error: extracted mean values were perturbed by random noise (σ = 0.05 kg) across 1,000 simulations, and the model refitted each time. 95% of estimates for A lay between 74.2 and 76.6%, and for k between 0.029 and 0.031 per week. These results suggest the effect of digitisation error was negligible.

**E. Leave-one-study-out analysis**

We performed a leave-one-study-out analysis to assess the stability of our results on the estimated parameters *A* and *k*. Results indicated that fitted parameters were stable (figures 11 and S12).


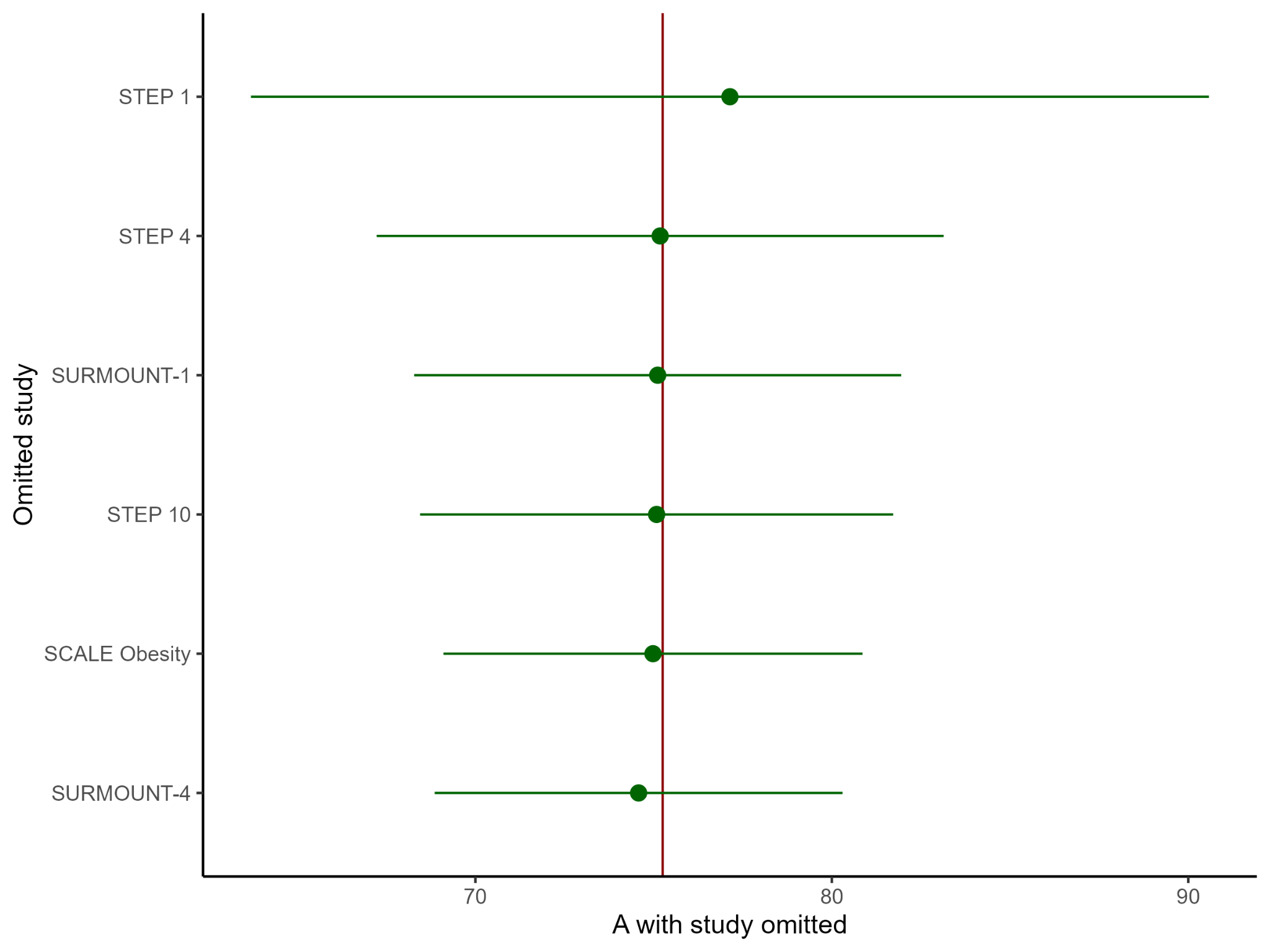
**Figure S11. Leave-one-study-out analysis showing fitted values of *A* with each study omitted.** Error bars represent 95% confidence intervals.


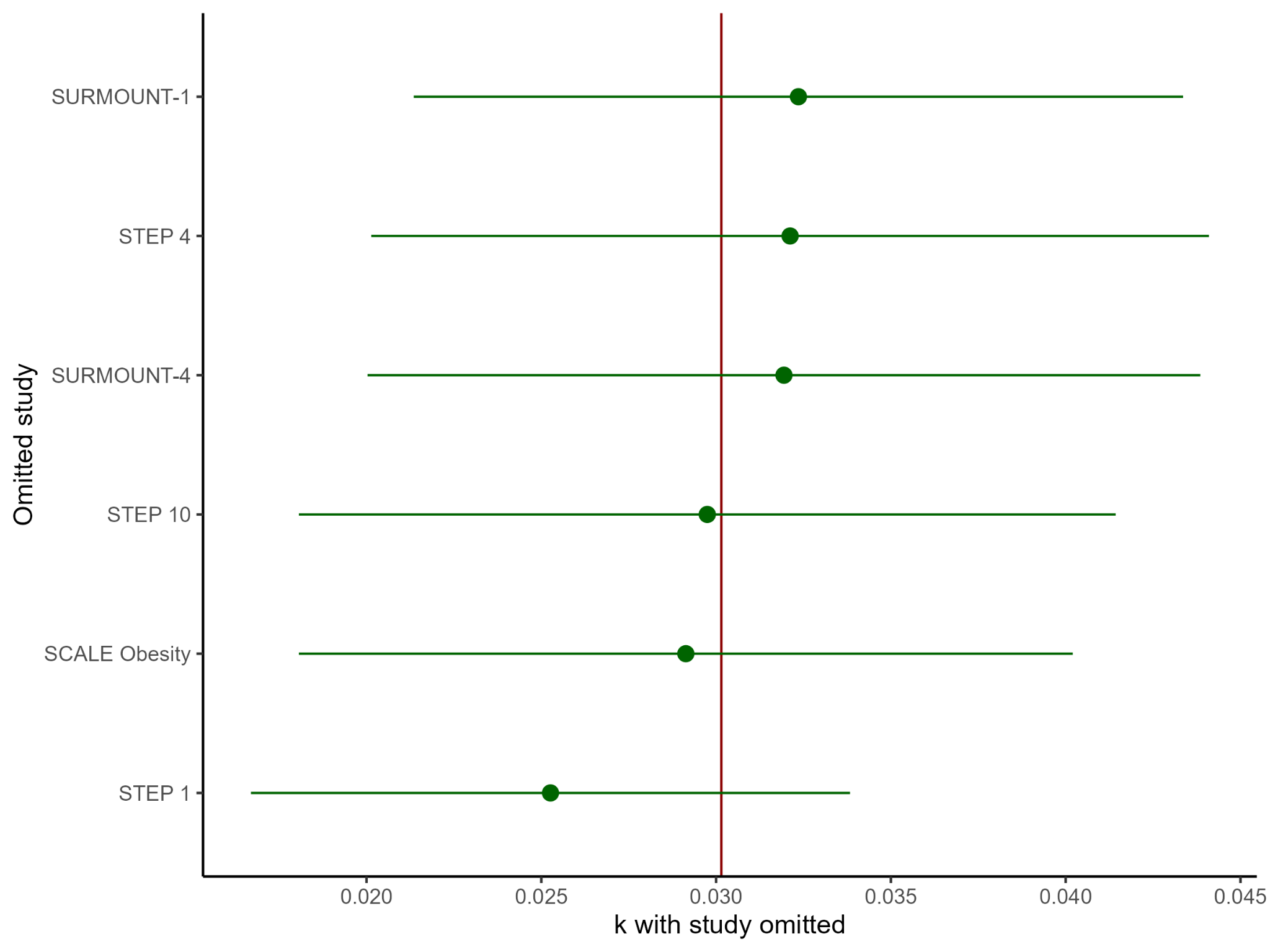


**Figure S12. Leave-one-study-out analysis showing fitted values of *k* with each study omitted.** Error bars represent 95% confidence intervals.

**F. Parametric bootstrap analysis**

Parametric bootstrapping (1000 replicates) was used to derive confidence intervals for the fixed effects of A and k. Bootstrapped 95% CIs (A = 70.5-88.1%, k = 0.0193–0.0391 per week) were similar to approximate Wald-type intervals (A = 68.9-81.6%, k = 0.0202–0.0401 per week).

**G. Study-level prediction band**

We generated a 95% prediction band (figure S13) based on the between-study standard deviation (τ) of the random effects on *k*, representing the expected band in which results from similar studies would likely fall.


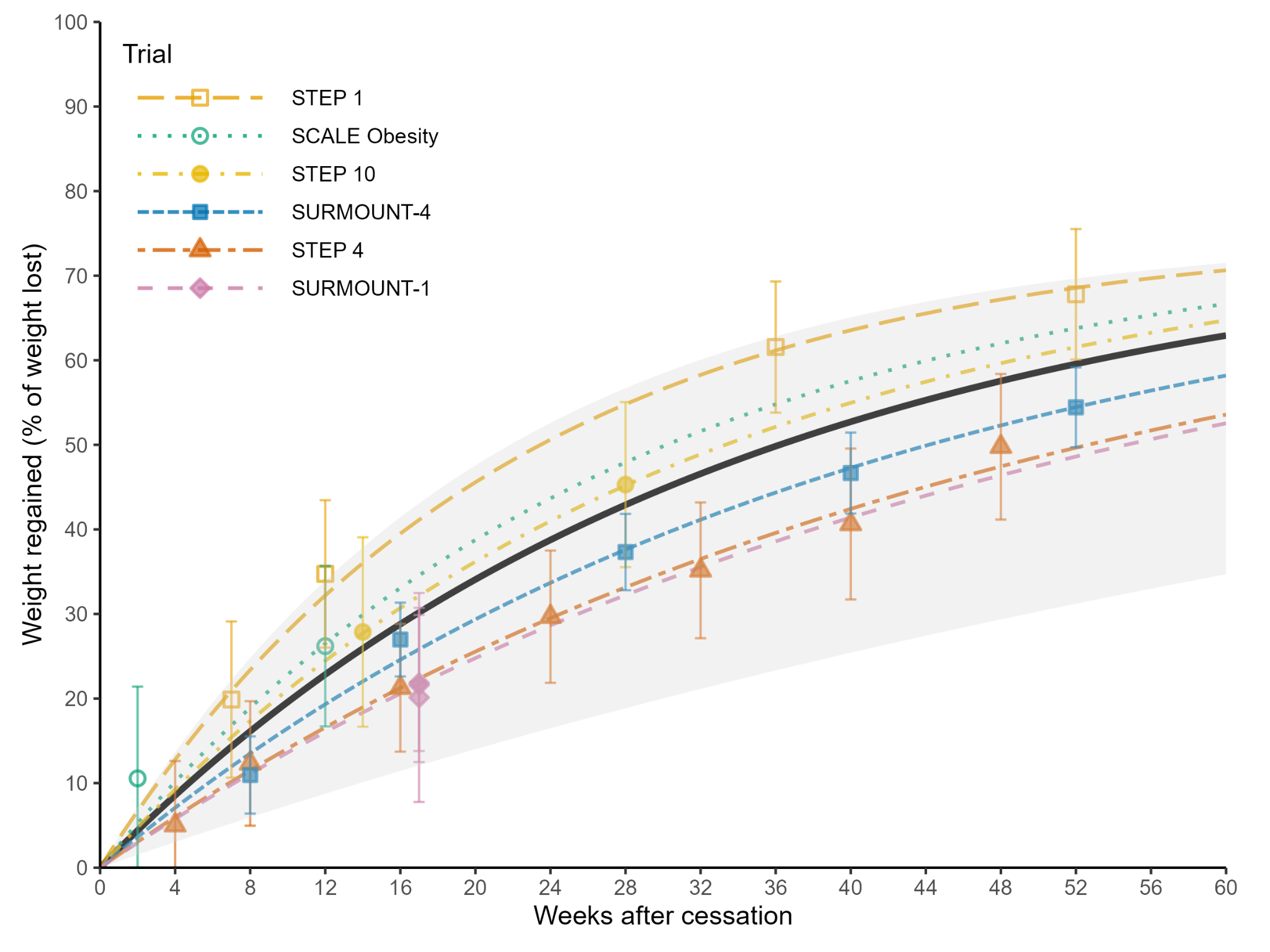


**Figure S13. 95% prediction band for study-level random effects on *k*, indicating where similar studies would be expected to fall.**

**H. Meta-analysis forest plots**

We conducted a conventional meta-analysis of percentage weight regain at 12, 26 and 52 weeks (including data points within ±2, ±2 and ±4 weeks respectively). Forest plots are shown in figure S14. Mean values were broadly consistent with our exponential recovery model.


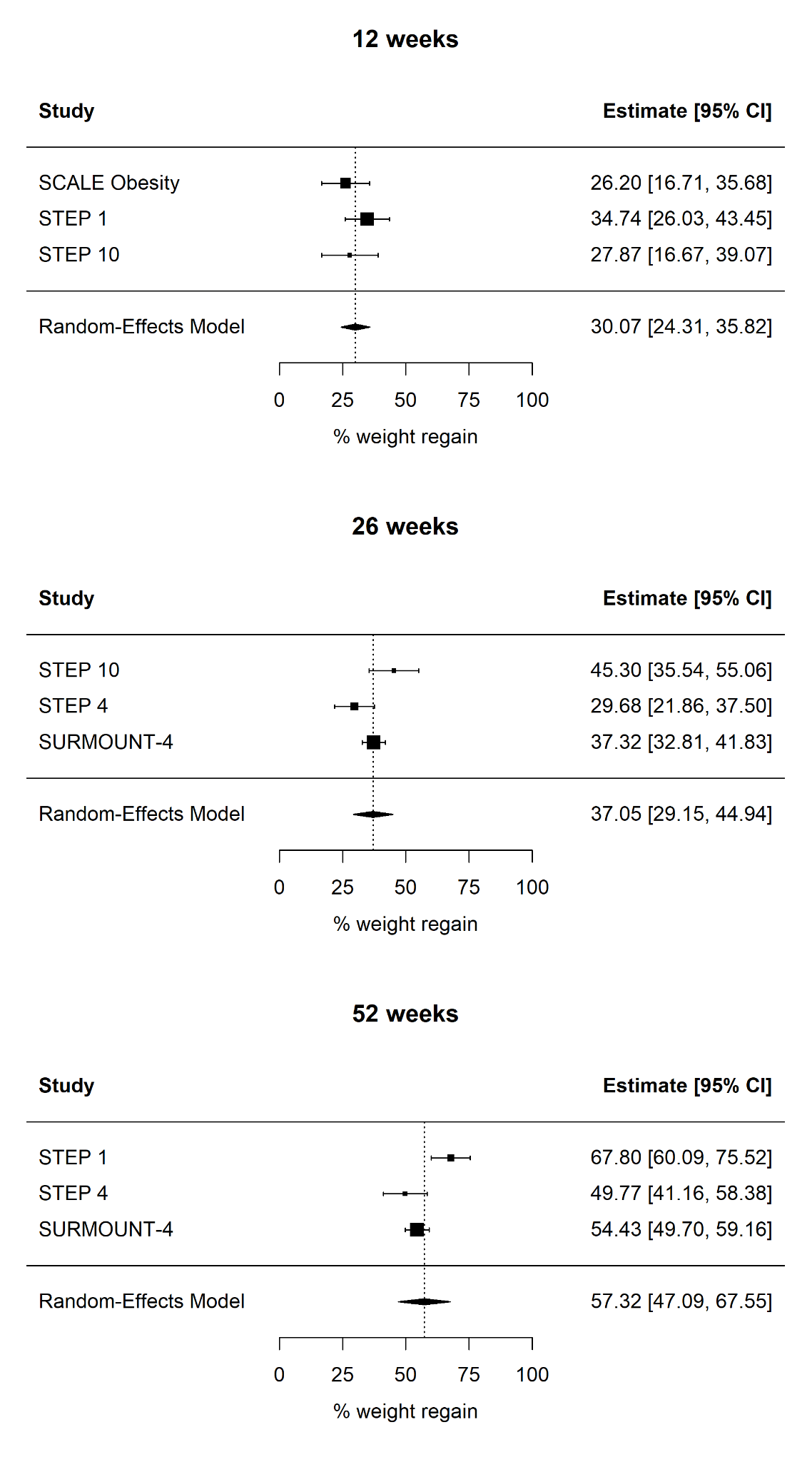


**Figure S14. Forest plots of percentage weight regain at 12, 26 and 52 weeks, including data points within ±2, ±2 and ±4 weeks respectively.**

# 5. GRADE Summary of findings

Table S3. Summary of findings table

| **Outcome** | **No. of studies** | **Study design** | **Risk of bias** | **Inconsistency** | **Indirectness** | **Imprecision** | **Estimated parameters** | | **Certainty of the evidence** |
| --- | --- | --- | --- | --- | --- | --- | --- | --- | --- |
|  |  |  |  |  |  |  | ***A (maximum weight regain)*** | ***k* (weight recovery rate constant)** |  |
| Weight regain | 6 | RCTs | Low | No concern | No concern | No concern | **75.3% (95% CI 68.9-81.6)** | **0.0302 per week (95% CI 0.0202-0.0401)** | ⊕⊕⊕⊕  **High** |

#

# References

[1.](https://www.zotero.org/google-docs/?PvrecE)  [Altintas Dogan AD, Hilberg O, Hess S, Jensen TT, Bladbjerg EM, Juhl CB. Respiratory Effects of Treatment with a Glucagon-Like Peptide-1 Receptor Agonist in Patients Suffering from Obesity and Chronic Obstructive Pulmonary Disease. Int J Chron Obstruct Pulmon Dis. 2022 Feb;Volume 17:405–14.](https://www.zotero.org/google-docs/?PvrecE)

[2.](https://www.zotero.org/google-docs/?PvrecE)  [Apperloo EM, Gorriz JL, Soler MJ, Cigarrán Guldris S, Cruzado JM, Puchades MJ, et al. Semaglutide in patients with overweight or obesity and chronic kidney disease without diabetes: a randomized double-blind placebo-controlled clinical trial. Nat Med. 2025 Jan;31(1):278–85.](https://www.zotero.org/google-docs/?PvrecE)

[3.](https://www.zotero.org/google-docs/?PvrecE)  [Armstrong MJ, Gaunt P, Aithal GP, Barton D, Hull D, Parker R, et al. Liraglutide safety and efficacy in patients with non-alcoholic steatohepatitis (LEAN): a multicentre, double-blind, randomised, placebo-controlled phase 2 study. The Lancet. 2016 Feb 13;387(10019):679–90.](https://www.zotero.org/google-docs/?PvrecE)

[4.](https://www.zotero.org/google-docs/?PvrecE)  [Aronne LJ, Sattar N, Horn DB, Bays HE, Wharton S, Lin WY, et al. Continued Treatment With Tirzepatide for Maintenance of Weight Reduction in Adults With Obesity: The SURMOUNT-4 Randomized Clinical Trial. JAMA. 2024 Jan 2;331(1):38–48.](https://www.zotero.org/google-docs/?PvrecE)

[5.](https://www.zotero.org/google-docs/?PvrecE)  [Asano M, Sekikawa A, Sugeno M, Matsuoka O, Robertson D, Hansen L. Safety/tolerability, efficacy and pharmacokinetics of 600-μg cotadutide in Japanese type 2 diabetes patients with a body mass index of 25 kg/m2 or higher: A phase I, randomized, double-blind, placebo-controlled study. Diabetes Obes Metab. 2023 Aug;25(8):2290–9.](https://www.zotero.org/google-docs/?PvrecE)

[6.](https://www.zotero.org/google-docs/?PvrecE)  [Barnett AH, Burger J, Johns D, Brodows R, Kendall DM, Roberts A, et al. Tolerability and efficacy of exenatide and titrated insulin glargine in adult patients with type 2 diabetes previously uncontrolled with metformin or a sulfonylurea: A multinational, randomized, open-label, two-period, crossover noninferiority trial. Clin Ther. 2007 Nov 1;29(11):2333–48.](https://www.zotero.org/google-docs/?PvrecE)

[7.](https://www.zotero.org/google-docs/?PvrecE)  [Bartelt K, Mast C, Deckert J, Gracianette M, Joyce B. Many Patients Maintain Weight Loss a Year After Stopping Semaglutide and Liraglutide. 2024 Jan 23 [cited 2025 June 7]; Available from: https://epicresearch.org/articles/many-patients-maintain-weight-loss-a-year-after-stopping-semaglutide-and-liraglutide](https://www.zotero.org/google-docs/?PvrecE)

[8.](https://www.zotero.org/google-docs/?PvrecE)  [Bunck MC, Cornér A, Eliasson B, Heine RJ, Shaginian RM, Taskinen MR, et al. Effects of Exenatide on Measures of β-Cell Function After 3 Years in Metformin-Treated Patients With Type 2 Diabetes. Diabetes Care. 2011 Aug 19;34(9):2041–7.](https://www.zotero.org/google-docs/?PvrecE)

[9.](https://www.zotero.org/google-docs/?PvrecE)  [Chen K, Chen L, Shan Z, Wang G, Qu S, Qin G, et al. Beinaglutide for weight management in Chinese individuals with overweight or obesity: A phase 3 randomized controlled clinical study. Diabetes Obes Metab. 2024 Feb;26(2):690–8.](https://www.zotero.org/google-docs/?PvrecE)

[10.](https://www.zotero.org/google-docs/?PvrecE)  [D’Alessio D, Häring HU, Charbonnel B, de Pablos-Velasco P, Candelas C, Dain MP, et al. Comparison of insulin glargine and liraglutide added to oral agents in patients with poorly controlled type 2 diabetes. Diabetes Obes Metab. 2015 Feb;17(2):170–8.](https://www.zotero.org/google-docs/?PvrecE)

[11.](https://www.zotero.org/google-docs/?PvrecE)  [Davies MJ, Bergenstal R, Bode B, Kushner RF, Lewin A, Skjøth TV, et al. Efficacy of Liraglutide for Weight Loss Among Patients With Type 2 Diabetes: The SCALE Diabetes Randomized Clinical Trial. JAMA. 2015 Aug 18;314(7):687–99.](https://www.zotero.org/google-docs/?PvrecE)

[12.](https://www.zotero.org/google-docs/?PvrecE)  [Dusilová T, Kovář J, Laňková I, Thieme L, Hubáčková M, Šedivý P, et al. Semaglutide Treatment Effects on Liver Fat Content in Obese Subjects with Metabolic-Associated Steatotic Liver Disease (MASLD). J Clin Med. 2024 Jan;13(20):6100.](https://www.zotero.org/google-docs/?PvrecE)

[13.](https://www.zotero.org/google-docs/?PvrecE)  [Enebo LB, Berthelsen KK, Kankam M, Lund MT, Rubino DM, Satylganova A, et al. Safety, tolerability, pharmacokinetics, and pharmacodynamics of concomitant administration of multiple doses of cagrilintide with semaglutide 2·4 mg for weight management: a randomised, controlled, phase 1b trial. The Lancet. 2021 May 8;397(10286):1736–48.](https://www.zotero.org/google-docs/?PvrecE)

[14.](https://www.zotero.org/google-docs/?PvrecE)  [Ferjan S, Janez A, Jensterle M. Dipeptidyl Peptidase-4 Inhibitor Sitagliptin Prevented Weight Regain in Obese Women with Polycystic Ovary Syndrome Previously Treated with Liraglutide: A Pilot Randomized Study. Metab Syndr Relat Disord. 2017 Dec;15(10):515–20.](https://www.zotero.org/google-docs/?PvrecE)

[15.](https://www.zotero.org/google-docs/?PvrecE)  [Ferrari F, Fierabracci P, Salvetti G, Jaccheri R, Vitti J, Scartabelli G, et al. Weight loss effect of liraglutide in real-life: the experience of a single Italian obesity center. J Endocrinol Invest. 2020 Dec 1;43(12):1779–85.](https://www.zotero.org/google-docs/?PvrecE)

[16.](https://www.zotero.org/google-docs/?PvrecE)  [Fineman M, Flanagan S, Taylor K, Aisporna M, Shen LZ, Mace KF, et al. Pharmacokinetics and Pharmacodynamics of Exenatide Extended-Release After Single and Multiple Dosing. Clin Pharmacokinet. 2011 Jan 1;50(1):65–74.](https://www.zotero.org/google-docs/?PvrecE)

[17.](https://www.zotero.org/google-docs/?PvrecE)  [Frias JP, Choi J, Rosenstock J, Popescu L, Niemoeller E, Muehlen-Bartmer I, et al. Efficacy and Safety of Once-Weekly Efpeglenatide Monotherapy Versus Placebo in Type 2 Diabetes: The AMPLITUDE-M Randomized Controlled Trial. Diabetes Care. 2022 July 6;45(7):1592–600.](https://www.zotero.org/google-docs/?PvrecE)

[18.](https://www.zotero.org/google-docs/?PvrecE)  [Garcia de Lucas MD, Olalla Sierra J. Canagliflozin as a replacement therapy for patients with type 2 diabetes not responding to GLP-1 receptor agonists. Diabetes Metab. 2017 Sept 1;43(4):373–4.](https://www.zotero.org/google-docs/?PvrecE)

[19.](https://www.zotero.org/google-docs/?PvrecE)  [Gibbons C, Blundell J, Tetens Hoff S, Dahl K, Bauer R, Bækdal T. Effects of oral semaglutide on energy intake, food preference, appetite, control of eating and body weight in subjects with type 2 diabetes. Diabetes Obes Metab. 2021 Feb;23(2):581–8.](https://www.zotero.org/google-docs/?PvrecE)

[20.](https://www.zotero.org/google-docs/?PvrecE)  [Jastreboff AM, Roux CW le, Stefanski A, Aronne LJ, Halpern B, Wharton S, et al. Tirzepatide for Obesity Treatment and Diabetes Prevention. N Engl J Med. 2025 Mar 5;392(10):958–71.](https://www.zotero.org/google-docs/?PvrecE)

[21.](https://www.zotero.org/google-docs/?PvrecE)  [Jensen SBK, Blond MB, Sandsdal RM, Olsen LM, Juhl CR, Lundgren JR, et al. Healthy weight loss maintenance with exercise, GLP-1 receptor agonist, or both combined followed by one year without treatment: a post-treatment analysis of a randomised placebo-controlled trial. eClinicalMedicine [Internet]. 2024 Mar 1 [cited 2025 June 7];69. Available from: https://www.thelancet.com/journals/eclinm/article/PIIS2589-5370(24)00054-3/fulltext](https://www.zotero.org/google-docs/?PvrecE)

[22.](https://www.zotero.org/google-docs/?PvrecE)  [Jensterle M, Ferjan S, Janez A. The maintenance of long-term weight loss after semaglutide withdrawal in obese women with PCOS treated with metformin: a 2-year observational study. Front Endocrinol [Internet]. 2024 Apr 11 [cited 2025 June 8];15. Available from: https://www.frontiersin.org/journals/endocrinology/articles/10.3389/fendo.2024.1366940/full](https://www.zotero.org/google-docs/?PvrecE)

[23.](https://www.zotero.org/google-docs/?PvrecE)  [Ji L, Jiang H, Cheng Z, Qiu W, Liao L, Zhang Y, et al. A phase 2 randomised controlled trial of mazdutide in Chinese overweight adults or adults with obesity. Nat Commun. 2023 Dec 14;14(1):8289.](https://www.zotero.org/google-docs/?PvrecE)

[24.](https://www.zotero.org/google-docs/?PvrecE)  [Ji L, Gao L, Xue H, Tian J, Wang K, Jiang H, et al. Efficacy and safety of a biased GLP-1 receptor agonist ecnoglutide in adults with overweight or obesity: a multicentre, randomised, double-blind, placebo-controlled, phase 3 trial. Lancet Diabetes Endocrinol. 2025 Sept 1;13(9):777–89.](https://www.zotero.org/google-docs/?PvrecE)

[25.](https://www.zotero.org/google-docs/?PvrecE)  [Ji L, Jiang H, Bi Y, Li H, Tian J, Liu D, et al. Once-Weekly Mazdutide in Chinese Adults with Obesity or Overweight. N Engl J Med. 2025 June 11;392(22):2215–25.](https://www.zotero.org/google-docs/?PvrecE)

[26.](https://www.zotero.org/google-docs/?PvrecE)  [Khoo J, Hsiang JC, Taneja R, Koo SH, Soon GH, Kam CJ, et al. Randomized trial comparing effects of weight loss by liraglutide with lifestyle modification in non-alcoholic fatty liver disease. Liver Int. 2019;39(5):941–9.](https://www.zotero.org/google-docs/?PvrecE)

[27.](https://www.zotero.org/google-docs/?PvrecE)  [Kubota M, Yamamoto K, Yoshiyama S. Effect on Hemoglobin A1c (HbA1c) and Body Weight After Discontinuation of Tirzepatide, a Novel Glucose-Dependent Insulinotropic Peptide (GIP) and Glucagon-Like Peptide-1 (GLP-1) Receptor Agonist: A Single-Center Case Series Study. Cureus [Internet]. 2023 Oct 4 [cited 2025 June 8];15(10). Available from: https://www.cureus.com/articles/191477-effect-on-hemoglobin-a1c-hba1c-and-body-weight-after-discontinuation-of-tirzepatide-a-novel-glucose-dependent-insulinotropic-peptide-gip-and-glucagon-like-peptide-1-glp-1-receptor-agonist-a-single-center-case-series-study](https://www.zotero.org/google-docs/?PvrecE)

[28.](https://www.zotero.org/google-docs/?PvrecE)  [Lau DCW, Erichsen L, Francisco AM, Satylganova A, Le Roux CW, McGowan B, et al. Once-weekly cagrilintide for weight management in people with overweight and obesity: a multicentre, randomised, double-blind, placebo-controlled and active-controlled, dose-finding phase 2 trial. The Lancet. 2021 Dec;398(10317):2160–72.](https://www.zotero.org/google-docs/?PvrecE)

[29.](https://www.zotero.org/google-docs/?PvrecE)  [le Roux CW, Astrup A, Fujioka K, Greenway F, Lau DCW, Van Gaal L, et al. 3 years of liraglutide versus placebo for type 2 diabetes risk reduction and weight management in individuals with prediabetes: a randomised, double-blind trial. The Lancet. 2017 Apr 8;389(10077):1399–409.](https://www.zotero.org/google-docs/?PvrecE)

[30.](https://www.zotero.org/google-docs/?PvrecE)  [McGowan BM, Bruun JM, Capehorn M, Pedersen SD, Pietiläinen KH, Muniraju HAK, et al. Efficacy and safety of once-weekly semaglutide 2·4 mg versus placebo in people with obesity and prediabetes (STEP 10): a randomised, double-blind, placebo-controlled, multicentre phase 3 trial. Lancet Diabetes Endocrinol. 2024 Sept 1;12(9):631–42.](https://www.zotero.org/google-docs/?PvrecE)

[31.](https://www.zotero.org/google-docs/?PvrecE)  [McInnes N, Hall S, Lochnan HA, Harris SB, Punthakee Z, Sigal RJ, et al. Diabetes remission and relapse following an intensive metabolic intervention combining insulin glargine/lixisenatide, metformin and lifestyle approaches: Results of a randomised controlled trial. Diabetes Obes Metab. 2023 Nov;25(11):3347–55.](https://www.zotero.org/google-docs/?PvrecE)

[32.](https://www.zotero.org/google-docs/?PvrecE)  [McKenzie AL, Athinarayanan SJ. Impact of Glucagon-Like Peptide 1 Agonist Deprescription in Type 2 Diabetes in a Real-World Setting: A Propensity Score Matched Cohort Study. Diabetes Ther. 2024 Apr 1;15(4):843–53.](https://www.zotero.org/google-docs/?PvrecE)

[33.](https://www.zotero.org/google-docs/?PvrecE)  [Montvida O, Klein K, Kumar S, Khunti K, Paul SK. Addition of or switch to insulin therapy in people treated with glucagon-like peptide-1 receptor agonists: A real-world study in 66 583 patients. Diabetes Obes Metab. 2017 Jan;19(1):108–17.](https://www.zotero.org/google-docs/?PvrecE)

[34.](https://www.zotero.org/google-docs/?PvrecE)  [Moolla A, Poolman T, Othonos N, Dong J, Smith K, Cornfield T, et al. Randomised trial comparing weight loss through lifestyle and GLP-1 receptor agonist therapy in people with MASLD. JHEP Rep. 2025 May 1;7(5):101363.](https://www.zotero.org/google-docs/?PvrecE)

[35.](https://www.zotero.org/google-docs/?PvrecE)  [O’Neil PM, Birkenfeld AL, McGowan B, Mosenzon O, Pedersen SD, Wharton S, et al. Efficacy and safety of semaglutide compared with liraglutide and placebo for weight loss in patients with obesity: a randomised, double-blind, placebo and active controlled, dose-ranging, phase 2 trial. The Lancet. 2018 Aug 25;392(10148):637–49.](https://www.zotero.org/google-docs/?PvrecE)

[36.](https://www.zotero.org/google-docs/?PvrecE)  [Punthakee Z, Hall S, McInnes N, Sherifali D, Tsiplova K, Kirabo FR, et al. Evaluating remission of type 2 diabetes using a metabolic intervention including fixed-ratio insulin degludec and liraglutide: A randomized controlled trial. Diabetes Obes Metab. 2024;26(12):5600–8.](https://www.zotero.org/google-docs/?PvrecE)

[37.](https://www.zotero.org/google-docs/?PvrecE)  [Rosenstock J, Klaff LJ, Schwartz S, Northrup J, Holcombe JH, Wilhelm K, et al. Effects of Exenatide and Lifestyle Modification on Body Weight and Glucose Tolerance in Obese Subjects With and Without Pre-Diabetes. Diabetes Care. 2010 Mar 23;33(6):1173–5.](https://www.zotero.org/google-docs/?PvrecE)

[38.](https://www.zotero.org/google-docs/?PvrecE)  [Rubino D, Abrahamsson N, Davies M, Hesse D, Greenway FL, Jensen C, et al. Effect of Continued Weekly Subcutaneous Semaglutide vs Placebo on Weight Loss Maintenance in Adults With Overweight or Obesity: The STEP 4 Randomized Clinical Trial. JAMA. 2021 Apr 13;325(14):1414–25.](https://www.zotero.org/google-docs/?PvrecE)

[39.](https://www.zotero.org/google-docs/?PvrecE)  [Sanyal AJ, Kaplan LM, Frias JP, Brouwers B, Wu Q, Thomas MK, et al. Triple hormone receptor agonist retatrutide for metabolic dysfunction-associated steatotic liver disease: a randomized phase 2a trial. Nat Med. 2024 July;30(7):2037–48.](https://www.zotero.org/google-docs/?PvrecE)

[40.](https://www.zotero.org/google-docs/?PvrecE)  [Seier S, Stamp-Larsen K, Jensen SBK, Torekov SS, Gudbergsen H. Treat to Target in Weight Management with Semaglutide: Real-World Evidence from an eHealth Clinic [Internet]. Rochester, NY: Social Science Research Network; 2025 [cited 2025 June 7]. Available from: https://papers.ssrn.com/abstract=5207768](https://www.zotero.org/google-docs/?PvrecE)

[41.](https://www.zotero.org/google-docs/?PvrecE)  [Siskind D, Russell A, Gamble C, Baker A, Cosgrove P, Burton L, et al. Metabolic measures 12 months after a randomised controlled trial of treatment of clozapine associated obesity and diabetes with exenatide (CODEX). J Psychiatr Res. 2020 May 1;124:9–12.](https://www.zotero.org/google-docs/?PvrecE)

[42.](https://www.zotero.org/google-docs/?PvrecE)  [Svensson CK, Larsen JR, Vedtofte L, Jakobsen MSL, Jespersen HR, Jakobsen MI, et al. One-year follow-up on liraglutide treatment for prediabetes and overweight/obesity in clozapine- or olanzapine-treated patients. Acta Psychiatr Scand. 2019;139(1):26–36.](https://www.zotero.org/google-docs/?PvrecE)

[43.](https://www.zotero.org/google-docs/?PvrecE)  [Touzot M, Voican A, Potier L, Beaussier H, Cachanado M, Sacco E, et al. Efficacy and tolerance of liraglutide for weight loss in obese, type 2 diabetes and haemodialysis patients. Diabetes Obes Metab. 2025;27(8):4599–602.](https://www.zotero.org/google-docs/?PvrecE)

[44.](https://www.zotero.org/google-docs/?PvrecE)  [Varanasi A, Chaudhuri A, Dhindsa S, Arora A, Lohano T, Vora MR, et al. Durability of Effects of Exenatide Treatment on Glycemic Control, Body Weight, Systolic Blood Pressure, C-Reactive Protein, and Triglyceride Concentrations. Endocr Pract. 2011 Mar 1;17(2):192–200.](https://www.zotero.org/google-docs/?PvrecE)

[45.](https://www.zotero.org/google-docs/?PvrecE)  [Wadden TA, Hollander P, Klein S, Niswender K, Woo V, Hale PM, et al. Weight maintenance and additional weight loss with liraglutide after low-calorie-diet-induced weight loss: The SCALE Maintenance randomized study. Int J Obes. 2013 Nov;37(11):1443–51.](https://www.zotero.org/google-docs/?PvrecE)

[46.](https://www.zotero.org/google-docs/?PvrecE)  [Wilding JPH, Batterham RL, Davies M, Van Gaal LF, Kandler K, Konakli K, et al. Weight regain and cardiometabolic effects after withdrawal of semaglutide: The STEP 1 trial extension. Diabetes Obes Metab. 2022 Aug;24(8):1553–64.](https://www.zotero.org/google-docs/?PvrecE)

[47.](https://www.zotero.org/google-docs/?PvrecE)  [Yu J, Lee J, Lee SH, Cho JH, Kim HS. A Study on Weight Loss Cause as per the Side Effect of Liraglutide. Cardiovasc Ther. 2022;2022(1):5201684.](https://www.zotero.org/google-docs/?PvrecE)

[48.](https://www.zotero.org/google-docs/?PvrecE)  [Zhou F, Jiang L, Guo J, Fan Y, Pan Q, Li T, et al. Degree of obesity and gastrointestinal adverse reactions influence the weight loss effect of liraglutide in overweight or obese patients with type 2 diabetes. Ther Adv Chronic Dis. 2023;14:20406223231161516.](https://www.zotero.org/google-docs/?PvrecE)
